# Dicer Cooperates with p53 to Suppress DNA Damage and Skin Carcinogenesis in Mice

**DOI:** 10.1371/journal.pone.0100920

**Published:** 2014-06-30

**Authors:** Stephen Lyle, Kathleen Hoover, Cansu Colpan, Zhiqing Zhu, Zdenka Matijasevic, Stephen N. Jones

**Affiliations:** 1 Department of Cancer Biology, University of Massachusetts Medical School, Worcester, Massachusetts, United States of America; 2 Department of Cell and Developmental Biology, University of Massachusetts Medical School, Worcester, Massachusetts, United States of America; University of Connecticut Health Center, United States of America

## Abstract

Dicer is required for the maturation of microRNA, and loss of Dicer and miRNA processing has been found to alter numerous biological events during embryogenesis, including the development of mammalian skin and hair. We have previously examined the role of miRNA biogenesis in mouse embryonic fibroblasts and found that deletion of Dicer induces cell senescence regulated, in part, by the p53 tumor suppressor. Although Dicer and miRNA molecules are thought to have either oncogenic or tumor suppressing roles in various types of cancer, a role for Dicer and miRNAs in skin carcinogenesis has not been established. Here we show that perinatal ablation of Dicer in the skin of mice leads to loss of fur in adult mice, increased epidermal cell proliferation and apoptosis, and the accumulation of widespread DNA damage in epidermal cells. Co-ablation of Dicer and p53 did not alter the timing or extent of fur loss, but greatly reduced survival of Dicer-skin ablated mice, as these mice developed multiple and highly aggressive skin carcinomas. Our results describe a new mouse model for spontaneous basal and squamous cell tumorigenesis. Furthermore, our findings reveal that loss of Dicer in the epidermis induces extensive DNA damage, activation of the DNA damage response and p53-dependent apoptosis, and that Dicer and p53 cooperate to suppress mammalian skin carcinogenesis.

## Introduction

MicroRNAs (miRNA) are small (19–24 nucleotides) single-stranded RNA molecules that regulate the expression of messenger RNA. Encoded within the mammalian genome in co-expressed gene clusters or as single genes, miRNA are transcribed by RNA polymerase II to generate primary precursor miRNA (pri-miRNA) transcripts of up to several thousand nucleotides in length. The pri-miRNA molecules form a secondary stem loop structure in the nucleus that is cleaved into precursor miRNA (pre-miRNA) species by a complex bearing the RNAse III Drosha and DCGR8 cofactor. The smaller (∼70 nucleotide) pre-miRNA molecules are subsequently transported to the cytoplasm by the nuclear transport receptor Exportin-5 and RanGTP, where pre-miRNA molecules undergo final cleavage by Dicer, a ribonuclease III-like enzyme, to form the mature miRNA. The miRNA molecules then assemble into ribonucleoprotein silencing complexes (RISC) and guide the silencing complex to specific mRNA molecules [1]. A high degree of complementarity between the target mRNA and miRNA results in degradation of the mRNA. If complementarity between the target mRNA and the miRNA is less, then regulation of gene function is achieved via translational repression of the cognate mRNA [2].

Mature miRNAs regulate numerous cell growth processes, including proliferation, apoptosis, senescence, and transformation, and both oncogenic and tumor-suppressing miRNAs have been described [3]. However, the net biological effect of miRNA loss on cell growth appears to differ in a cell specific manner. Dicer has been found to promote cell proliferation in mouse ES cells [4], [5] and our lab has previously shown that conditional Dicer ablation in primary mouse embryonic fibroblasts (MEF) blocks cell growth by activating the p53 tumor suppressor and inducing cell senescence [6]. These findings indicate that miRNA biogenesis facilitates normal (non-transformed) cell growth. In contrast, many human cancers display generally reduced levels of Dicer expression [3] and heterozygous germline mutations of Dicer have been identified in families with pleuropulmonary blastoma-inherited cancer syndrome [7]. Further evidence for a role for miRNA in suppressing cancer has been obtained in mice, wherein haplo-insufficiency for Dicer reduces survival in a model of *Kras*-driven lung cancers [8], and a reduction in *Dicer* gene dosage induces high-grade serous carcinomas within the fallopian tubes of PTEN-conditional mice [9]. Thus, while total loss of Dicer function appears deleterious to normal cell growth, Dicer activity is not absolutely required for the growth of transformed cells, and perturbation of Dicer and miRNA maturation may facilitate tumorigenesis in some settings [10].

Analysis of genetically altered mice has also revealed that Dicer plays important roles in regulating tissue morphogenesis and organ homeostasis [11]–[17]. In skin, studies employing keratinocyte (K14)-driven ablation of miRNA biogenesis during embryogenesis have revealed the importance of miRNA biogenesis in proper epidermis and hair follicle formation [18]–[20]. K14-Cre mediated ablation of either *Dicer* or *Dcgr8* conditional alleles in skin progenitor cells at E14.5 led to the evagination of early-stage hair follicles into the epidermis, where they formed germ-like cysts that disrupted the epidermis. Newborn mice lacked mature miRNAs in their skin, displayed reduced cell growth and increased apoptosis in developing hair follicles, and increased proliferation in the interfollicular epidermis [18]. Once born, these pups failed to gain weight, and most died within a week of birth presumably due to issues related to dehydration and failure to thrive. In keeping with these reports, a recent study employing mice bearing conditional *Dicer* or *Drosha* alleles and a temporally-inducible Keratin15-promoter determined that induced postnatal ablation of either miRNA processing enzyme reduced the survival of rapidly proliferating matrix population of cells that normally form the hair shaft during the anagen phase of the hair cycle [21]. Apoptosis within the matrix population led to a dermal inflammatory response, hyper-proliferation of the interfollicular epidermis, and degradation of the hair follicle.

The increase in epidermal cell proliferation seen after loss of miRNA biogenesis [18], [21] suggests that Dicer might function to suppress epithelial cell proliferation and skin carcinogenesis in mice. Although changes in mature miRNA expression patterns and in overall *Dicer* expression levels in melanoma and non-melanoma skin cancer patient biopsies have been observed [22]–[24], direct evidence of a role for miRNA biogenesis in skin cancer is lacking.

We have previously generated *Dicer* conditional (Dicer^c/c^) mice and analyzed the effects of Dicer ablation on cell growth [6]. Dicer ablation in MEFs induced DNA damage, stabilization and activation of the p53 tumor suppressor, and a rapid onset of p53-mediated cell senescence. Senescence was also observed *in vivo* in the follicular epithelium of young mice specifically deleted for Dicer in skin epithelium [6]. However, in contrast to the previous K14-Cre; Dicer^c/c^ or K14-Cre; DCGR8^c/c^ mouse studies [18]–[20], we employed a Keratin5-Cre transgene (K5-Cre+) that induced Dicer ablation in the epithelium of perinatal stage mice (E17.5- P0). Interestingly, K5-Cre+, Dicer^c/c^ mice lacking *Dicer* expression in the epidermis (Dicer^Δ/Δ^) survived the postnatal period. In this present study, we now utilize this K5-Cre+, Dicer^c/c^ model to examine the relative contributions of Dicer and p53 in epidermal carcinogenesis. Dicer^Δ/Δ^ mice survive to adulthood, and develop a normal fur coat that is subsequently lost at 2-3 months of age. These mice have reduced and highly dysmorphic hair follicles, and the epidermis displays increased cell proliferation, apoptosis, a large amount of DNA damage, and activation of the DNA damage response. Co-deletion of p53 does not alter the follicle dysmorphology and fur loss in adult Dicer^Δ/Δ^ mice, but promotes cell proliferation, reduces apoptosis, and leads to the formation of multiple and poorly differentiated squamous cell carcinomas and basal cell carcinomas. These results reveal that Dicer cooperates with p53 to suppress mammalian skin carcinogenesis.

## Materials and Methods

### Ethics statement

Mice were utilized in strict accordance with federal guidelines and those established by the University of Massachusetts Medical School. This study was specifically approved by the University of Massachusetts Medical School's Institutional Animal Care and Use Committee (Docket A1026).

### Mouse and tissue genotyping

The generation of the Dicer conditional mouse model [6] and the use of K5-Cre transgenic mice [6], [25] to delete conditional alleles specifically in the epidermis have been described previously. All experimental and littermate control mice used in this study were on a mixed 129Sv X C57Bl/6N background. Genomic DNA PCR was used to identify inheritance of the K5-Cre transgene (Cre forward primer 5′-CGGTCGATGCAACGAGTGAT-3′ and Cre reverse primer 5′-CCACCG TCAGTACGTGAGAT-3′) and to verify Dicer allelic excision (Dicer forward primers 5′- CCATTGGTGCCAAGACAATG-3′ and 5′-CCAAGATGCAGTGATCATTCC-3′ and Dicer reverse primer 5′–CAGGCTCCACTCCTAAC-3′). Genotyping of p53 wildtype, conditional, and ablated alleles was performed as described previously [26]. All mice were maintained in the UMMS vivarium employing standard (specific pathogen-free) husbandry.

### Fur loss in mice

A 3-cm^2^ square area on the mid-dorsal region of the mouse was assayed every 2-3 days for loss of fur. Fur loss was scored every 2-4 days in six Dicer^Δ/Δ^ mice, seven Dicer^Δ/Δ^, p53^wt/Δ^ mice, and six Dicer^Δ/Δ^, p53^Δ/Δ^, mice as the percentage of the square area per mouse that retained hair.

### Histology

The fur (when present) was shaved from the right flank of the mice, and skin tissue harvested by dissection. Skin tissue was fixed in 10% formalin overnight. Skin sections (5 µm) were stained with hematoxylin and eosin (H&E) as performed by the UMMS DERC Morphology Core. Antibodies against phospho-S139 H2AX (at 1∶100 dilution), phospho-Ser1981 ATM (at 1∶100 dilution), and Ki-67 (at 1∶200 dilution) were purchased from Cell Signaling Technology (Danvers, MA). Images were digitally captured using a Zeiss camera system mounted on a Zeiss stage scope. Epidermal thickness was measured on H&E stained sections in micrometers: two representative measurements per animal and eight animals per genotype. The number of mitotic cells and apoptotic events were counted in 10 representative high power (40X) fields, eight animals per genotype. Asterisks presented above the histograph bars indicate p values<0.01 (unpaired t-test).

### Survival and Tumor assays

During the survival and spontaneous tumor studies, mice were inspected every other day for any signs of morbidity or for obvious tumor formation. Necropsies were performed on all mice at the time of death or when tumors were harvested, and selected tissues were isolated and fixed in 10% phosphate-buffered formalin overnight. Embedding, tissue sectioning, and H&E staining of the slides was performed by the UMMS DERC Morphology Core. Tumors were classified my morphology without knowledge of their genotypes. Statistical analysis and p values (Log-Rank test) were performed using STATA 12.0 software (College Station, TX)

## Results

We have previously generated K5-Cre+, Dicer^c/c^ mice [6]. Paternal inheritance of the K5-Cre+ in mice homozygous for Dicer-conditional alleles results in genetic ablation of Dicer specifically in the skin and stratified epithelium of newborn mice [27]. Dicer^c/Δ^ mice were indistinguishable at birth from K5-Cre+ mice, or from Dicer^c/c^ or Dicer^c/Δ^ littermates that failed to inherit the K5-Cre+ transgene. However, K5-Cre+ ablation of *Dicer* induced fur loss in Dicer^Δ/Δ^ mice shortly after weaning.

**Figure 1:**
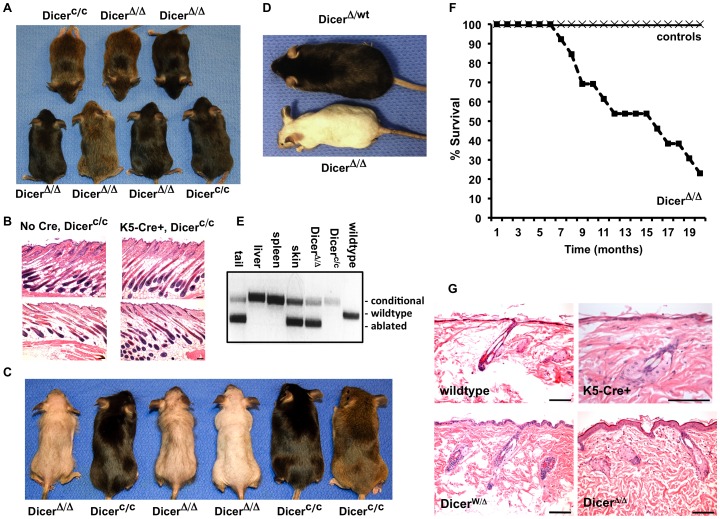
Ablation of *Dicer* by the K5-Cre transgene induces fur loss and epidermal dysmorphology in older mice. **A**) Indistinguishable appearance of *Dicer*-conditional littermates with (Dicer^Δ/Δ^) or without (Dicer^c/c^) the K5-Cre transgene at four weeks of age. **B**) Normal morphology of skin and hair follicles in four-week old control mice (Dicer^c/c^ mice, left two panels) and age-matched Dicer^Δ/Δ^ mice (Dicer^c/c^, K5Cre+, right two panels). Scale bars equal 100 microns. **C**) Appearance of litter of K5-Cre negative (Dicer^c/c^) and K5-Cre+ (Dicer^Δ/Δ^) mice at 10 weeks of age. **D**) Appearance of 8-month old Dicer heterozygous mouse (top) and Dicer-ablated mouse (bottom) Haplo-insufficieny for Dicer does not alter the fur of mice. **E**) PCR analysis of tissue-derived genomic DNA samples from representative K5Cre+, Dicer^c/c^ (Dicer^Δ/Δ^) mouse indicates presence of *Dicer*-ablated allele (283 bp product) in the tail and skin of the mouse, but not in the liver or spleen. PCR product of Dicer conditional allele and Dicer wildtype allele = 476 bp and 396 bp, respectively. Three control genomic DNA samples (Dicer^c/c^; K5Cre+, Dicer^c/c^; and wildtype) were used as controls. **F**) Kaplan-Meier survival curves for control (wildytype or Dicer^c/c^) cohorts and Dicer^Δ/Δ^ mouse cohorts. **G**) Hematoxylin and eosin staining of skin tissue sections of 7-month old mice. Deletion of *Dicer* in the epidermis (Dicer^Δ/Δ^ mice-bottom panels) results in defective follicle morphology. Wildtype (wt) and K5-Cre+ skin samples are used as controls (top panels). Scale bars equal 100 microns.

To further document the effects of *Dicer* ablation in the skin of mice, cohorts of Dicer^c/c^ mice that either lacked or contained the K5-Cre+ transgene (Dicer^Δ/Δ^) were collected and aged. At 4 weeks of age, Dicer^c/c^ mice and Dicer^Δ/Δ^ mice were of equal size and weight, and displayed fully developed fur coats (Fig. 1A). No morphological differences were detected in the skin of Dicer^c/c^ mice and Dicer^Δ/Δ^ mice at 4 weeks of age (Fig. 1B). However, Dicer^Δ/Δ^ mice began to lose their fur and whiskers shortly after the fifth week of age and retained only small patches of fur and a few misshapen whiskers by 10 weeks of age (Fig. 1C). In contrast, mice bearing a single ablated allele of Dicer and one non-ablated (wildtype) Dicer allele retain their fur and whiskers throughout their life (Fig.1D). Thus, bi-allelic deletion of Dicer induces fur loss in juvenile mice. As expected, the presence of the *Dicer*-ablation allele was observed specifically in the skin and tail of K5-Cre+, Dicer^c/c^ mice (Fig.1E), as activation of the keratin5 promoter in these tissues induces Cre-mediated deletion of the floxed alleles. Although non- ablated Dicer-conditional alleles can be detected in the skin using this PCR-based assay, this is most likely due to the presence of non-deleted Dicer alleles in keratinocytes, adipocytes, and other non-Cre expressing cells in the dermis and not due to incomplete deletion of Dicer in the epidermis, since haplo-insufficiency of Dicer does not induce fur loss in mice (Fig. 1D). In addition, loss of fur was not observed in K5-Cre+ mice that were either wildtype (Dicer^wt/wt^) or heterozygous (Dicer^wt/c^) for *Dicer*, nor was fur loss seen in Dicer^c/c^ mice or Dicer^wt/c^ mice that lacked the K5-Cre transgene.

At 3 months of age, the Dicer^Δ/Δ^ mice displayed a roughened epidermis and were devoid of any fur and whiskers. Although skin integrity was maintained in Dicer^Δ/Δ^ mice, their long-term survival was compromised. Dicer^Δ/Δ^ mice died as early as 6 months of age, with 80% of the cohort dead by 20 months (Fig. 1F). In contrast, no K5-Cre+ mice either Dicer^wt/wt^ or Dicer^wt/c^ died during this time (controls). The cause of death of the Dicer^Δ/Δ^ mice is unclear. Older Dicer^Δ/Δ^ mice were of similar size as age-matched wildtype or Dicer^wt/Δ^ mice, and no aberrant behavior was noted. Although several of the Dicer^Δ/Δ^ mice displayed mild cachexia prior to death, the necropsies were unremarkable and no organ malformations or tumors were observed in any tissues. Histologic analysis of skin harvested from 7-8 month old Dicer^Δ/Δ^ mice or from age-matched wildtype mice and K5-Cre+, Dicer^wt/wt^ mice revealed that ablation of *Dicer* in the epidermis resulted in defective and reduced numbers of hair follicles (Fig. 1G -bottom right panel). The follicles were debris-laden and miss-oriented, and enlarged cells and apoptotic figures were observed surrounding the degraded follicle structure. In contrast, the epidermis and follicles of Dicer^Δ/wt^ control mice appeared normal (Fig. 1G -bottom left panel). The interfollicular epidermis in Dicer^Δ/Δ^ mice had an increase in cellularity, whereas the dermal papillae, adipose, muscle layers, fibroblasts and keratin deposition appeared normal in these mice.

**Figure 2:**
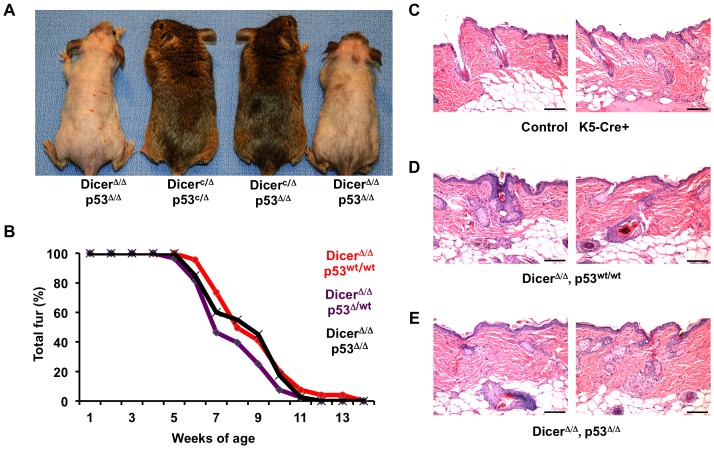
Co-ablation of p53 does not alter fur loss in Dicer^Δ/Δ^ mice. **A**) Appearance of mice at 5-months of age, with genotypes of the mice given beneath. As the two middle mice lack the K5-Cre transgene, not all conditional (c) alleles have undergone deletion. **B**) Fur loss was scored in *Dicer*-ablated mice bearing one (purple), two (red), or no (black) functional alleles of p53. Regardless of genotype, all Dicer^Δ/Δ^ mice began to lose fur between ages 5 and 6 weeks of age, and almost all fur is lost by week 12. **C**) Hematoxylin and eosin staining of skin tissue sections of two 8-week old K5-Cre transgenic control mice. **D**) Hematoxylin and eosin staining of skin tissue sections of two 8-week old Dicer skin-ablated mice. **E**) Hematoxylin and eosin staining of skin tissue sections of two 8-week old mice co-ablated in skin for Dicer and p53. Scale bars for 2C, 2D, and 2E equal 100 microns.

We have previously reported that upregulation of p53 activity in the epidermis of K5-Cre+, Mdm2^c/c^ mice promotes cell senescence and inhibits both epidermal function and fur regeneration [25]. To determine if alteration of p53 activity would affect the epidermal phenotype of Dicer^Δ/Δ^ mice, we intercrossed Dicer^c/c^ mice with mice bearing conditional alleles of p53 (p53^c/c^), and intercrossed the resulting compound heterozygous mice with K5-Cre+ Dicer^c/c^ mice to generate K5-Cre+, Dicer^c/c^ mice that had one or two conditional alleles of p53 [26]. Mice that were K5-Cre+, p53^c/c^ or K5-Cre+, p53^wt/c^ were generated as controls, as were mice that were hemizygous for *Dicer* and p53 or hemizygous for *Dicer* and ablated for p53. Paternal inheritance of the K5-Cre transgene in Dicer^c/c^ mice resulted in the loss of fur in Dicer^Δ/Δ^ mice co-ablated for p53 (Fig. 2A). In contrast, mice ablated solely for p53 in the skin (p53^Δ/Δ^) that retained one or both functional copies of *Dicer* retained a normal fur coat as they aged. The effect of p53 on the rate of fur loss was measured by counting individual hair follicles within a 3 cm^2^ area on the flank of Dicer^Δ/Δ^ mice that were either p53-wildtype (p53^wt/wt^), p53-heterozygous (p53^wtΔ^), or p53 co-ablated (p53^Δ/Δ^) in the epidermis (Fig. 2B). Regardless of p53 status, Dicer^Δ/Δ^ mice began to lose fur by the fifth week of age, and all mice were nearly devoid of fur and whiskers by twelve weeks. Skin samples were harvested from control K5-Cre transgenic mice and from representative Dicer-ablated mice that were either wildtype or co-ablated for p53 at eight weeks of age, the mid-point in time when the mice were undergoing fur loss. Control K5-Cre+ skin had normal numbers of follicles, with many follicles present in the telogen phase of the hair cycle (Fig. 2C). In contrast, the skin of Dicer-ablated mice displayed anagen growth phase hairs with dysmorphic follicles, abnormal hair shafts, and dyskertotic cells in the follicular epithelium (Fig. 2D). No morphologic difference was observed in Dicer-ablated skin that contained or lacked functional p53 (Fig. 2E).

**Figure 3:**
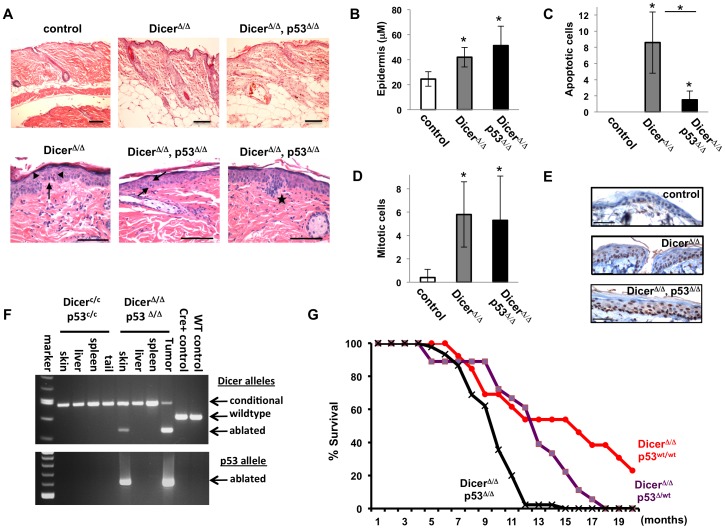
Co-ablation of p53 decreases apoptosis and the survival of Dicer^Δ/Δ^ mice. **A**) Hematoxylin and eosin staining of skin tissue sections of 5-month old mice. Control (K5-Cre+ transgene) was similar in appearance to wildtype control samples, whereas ablation of *Dicer* showed reduced and misshapen follicle morphology, with increased epithelial thickening, mitotic figures (arrow), and incidence of apoptosis (arrowheads). Ablation of p53 did not alter follicle dysmorphology seen in Dicer^Δ/Δ^ mice (upper right panel), though epithelial cellularity was more pronounced in some regions (see star on bottom right side panel) and fewer apoptotic events were noted. Scale bars equal 100 microns. B) Measurements of epidermal thickness in microns for control skin (white bar), Dicer-ablated skin (gray bar), and Dicer, p53 co-ablated skin (black bar). Bars represent average thickness, with standard deviation shown. Asterisks indicate p values<0.01 between control and Dicer-ablated skin or control and Dicer, p53 co-ablated skin. **C**) Average number of apoptotic cells observed in 40X field. No apoptotic figures were seen in control tissues (white bar) whereas apoptosis was increases in Dicer-ablated skin (gray bar) and in Dicer, p53 co-ablated skin (black bar) relative to controls. Apoptosis was decreased in Dicer-ablated skin co-deleted for functional p53. Asterisks indicate p values<0.01. **D**) Average number of mitotic figures seen in 40X field. Mitosis was increased in Dicer-ablated skin (gray bar) and in Dicer, p53 co-ablated skin (black bar) relative to controls (white bar). Asterisks indicate p values<0.01 between control and Dicer-ablated skin or control and Dicer, p53 co-ablated skin. **E**) Immuno-staining for the proliferation antigen Ki-67 confirms increased cell proliferation in the epidermis of *Dicer*-ablated mice in the presence or absence of functional *p53*. Scale bars equal 100 microns. **F**) PCR-based genotyping of tissues harvested from Dicer conditional, p53 conditional mice in the absence (Dicer^c/c^, p53^c/c^) or presence (Dicer^Δ/Δ^ p53^Δ/Δ^) of the K5-Cre transgene. Deletion of functional Dicer and p53 alleles is detected specifically in the skin and tumor tissues of K5-Cre+ mice. **G**) Kaplan-Meier survival curves for Dicer-ablated mice bearing one (purple; n = 17), two (red; n = 14), or no (black; n = 45) functional alleles of p53. Ablation of both *Dicer* and *p53* in the skin greatly reduced viability of these mice relative to mice ablated for *Dicer* in skin (p<0.01) or mice ablated for *Dicer* and one allele of *p53* in skin (p<0.01).

Histological analysis of the skin Dicer^Δ/Δ^ mice with or without functional p53 alleles at five months of age revealed that co-ablation of p53 also did not alter the follicle dysmorphology seen in Dicer^Δ/Δ^ mice in older mice (Fig. 3A -top panels). More mitotic cells (arrows) and apoptotic cells (arrowheads) were detected in Dicer-ablated skin than in control K5-Cre+ skin (Fig. 3A -bottom panels), and regions of increased cellularity with many mitotic figures were observed in the interfollicular epidermal layer and above the basal layer when Dicer and p53 were co-ablated. In addition, fewer apoptotic figures were present in co-ablated skin, and pre-malignant basaloid proliferation events were detected (Fig. 3A - bottom right panel- see star). There is also hypergranulosis, fewer apoptotic events, and some evidence of attempts to form new follicles in the Dicer^Δ/Δ^, p53^Δ/Δ^ skin compared to p53^wt/wt^ or p53^wt/Δ^ skin.

The increased cellularity in the epidermis of Dicer-ablated mice led to an increase in the thickness of the epidermis. Epidermal thickness was measured in control K5-Cre+ skin sections and in Dicer^Δ/Δ^ and Dicer^Δ/Δ^, p53^Δ/Δ^ skin sections (Fig. 3B). Control skin displayed a mean thickness of 24 micrometers, whereas Dicer^Δ/Δ^ and Dicer^Δ/Δ^, p53^Δ/Δ^ skin had a mean thickness of 42 micrometers and 51.3 micrometers, respectively. Interestingly, while no apoptotic cells were detected in control epidermis, there was a significant increase in the numbers of apoptotic cells in the epidermis when Dicer was ablated (Fig. 3C). This apoptosis was partly p53-dependent, as co-ablation of p53 greatly reduced the numbers of apoptotic events in the epidermis of Dicer-ablated mice. There was also an increase in the numbers of mitotic cells in Dicer-ablated epidermis, though the presence or absence of functional p53 alleles did not greatly alter the total number of mitotic events (Fig. 3D). This increase in cell proliferation was confirmed in adult mice by staining of skin sections for the proliferation antigen Ki-67 (Fig. 3E), as far more KI-67 positive cells were detected in Dicer-ablated epidermis (with or without functional p53) than in control epidermis.

To confirm Cre-mediated co-deletion of Dicer and p53 in the skin of mice, DNA was collected from representative 8-month old Dicer-conditional, p53-conditional mice either lacking the K5-Cre transgene (Dicer^c/c^, p53^c/c^) or bearing the K5-Cre transgene (Dicer^Δ/Δ^, p53^Δ/Δ^). PCR analysis of DNA confirmed no deletion of either the Dicer conditional allele or p53 conditional allele in representative organs of mice lacking the K5-Cre transgene, whereas Dicer^Δ/Δ^, p53^Δ/Δ^, mice bearing the K5-Cre transgene displayed Dicer and p53- ablated alleles in those tissues expressing Cre (Fig. 3F). Thus, the presence of deleted p53 alleles does not alter deletion of the Dicer allele in these tissues. Some non-deleted Dicer alleles were detected by the PCR assay in the skin and tumor tissue. As before (Fig. 1E), this is likely due to the presence of non-epidermal cells in the samples, as the Dicer^Δ/Δ^, p53^Δ/Δ^, mice are devoid of any fur whereas mice haplo-insufficient for Dicer retain fur regardless of p53 status (Fig. 2A).

Although reduced p53 gene dosage within the skin did not alter the fur loss in Dicer^Δ/Δ^ mice, it did impact their survival. Dicer^Δ/Δ^ mice either wildtype or haplo-insufficient for p53 in skin displayed a mean survival of 16 months or 13 months, respectively, whereas mice co-ablated for both Dicer and p53 in the skin lived an average of only 9 months. Although the reduced survival of K5-Cre+ Dicer^c/c^ mice with a single conditional allele of p53 is borderline significant (p =  0.067), ablation of both functional alleles of p53 greatly reduced survival of Dicer-ablated mice relative to Dicer-ablated, p53-wildtype (p = 0.0001) or Dicer-ablated, p53-haploinsufficient (p = 0.0002) mice. Furthermore, no Dicer^Δ/Δ^ mouse that was haplo-insufficient for p53 in skin survived beyond 18 months, whereas 20% of the Dicer^Δ/Δ^, p53-wildtype cohort was alive at 20 months of age (Fig. 3G). Thus, reduction in p53 gene dosage accelerated the demise of Dicer skin-ablated mice. The reason for increased lethality in Dicer^Δ/Δ^ mice deficient for functional p53 in the skin was due to the formation of highly aggressive and numerous skin tumors starting at 7-8 months of age.

**Figure 4:**
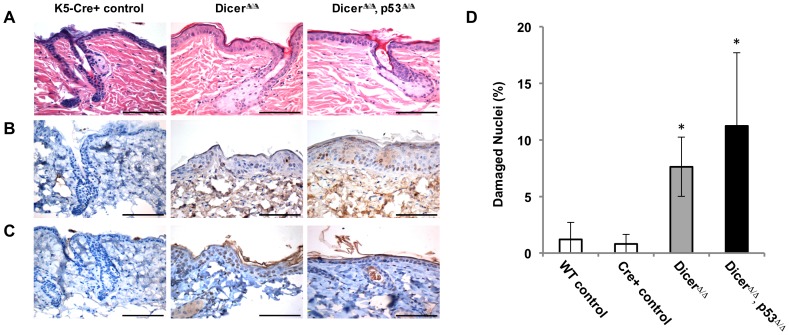
Deletion of Dicer in the epidermis induces DNA damage. **A**) Representative hematoxylin and eosin staining of skin sections of 7-8 month old K5-Cre+ control mice, and of 7-8 month old Dicer-ablated mice with (Dicer^Δ/Δ^) or without functional p53 (Dicer^Δ/Δ^, p53^Δ/Δ^). **B**) Phospho-H2A.X staining of representative skin samples reveals DNA damage in *Dicer*-ablated skin. **C**) Staining of sections for phospho-ATM indicates activation of DNA damage signaling (ATM auto-phosphorylation) specifically in *Dicer*-ablated skin, regardless of p53 status. Scale bars equal 100 microns. **D**) Quantification of DNA damage levels in the epidermis of Dicer-ablated mice. Average percentage of phospho-H2A.X positive nuclei shown (bars), with standard deviation. Little phospho-H2A.X staining was observed in the epidermis of wildtype mice or of K5-Cre control mice (white bars), whereas increased staining was seen in Dicer-ablated skin (gray bar) and in Dicer, p53 co-ablated skin (black bar) relative to controls. Asterisks indicate p values<0.01 between control and Dicer-ablated skin or control and Dicer, p53 co-ablated skin.

We have previously reported that loss of miRNA biogenesis can induce DNA damage in primary cells [6]. Therefore, we examined DNA damage in the epidermal tissues of 7-8 month old control mice (K5-Cre+, Dicer and p53-wildtype), or in age-matched mice that were ablated for Dicer alone or co-ablated for Dicer and p53 (Fig. 4). Phospho-H2A.X antibody staining was performed on these skin samples indicate that a large amount of DNA damage is present in the basal layer of follicular epithelium as well as in the interfollicular epidermis of Dicer^Δ/Δ^ mice (Fig. 4B). DNA damage was also readily detected in the skin of Dicer^Δ/Δ^, p53^Δ/Δ^ mice, both in normal epithelium and in areas of the epidermis appearing to undergo pre-neoplastic changes. To confirm that the DNA damage was triggering a DNA damage response, we also stained these tissues for activation of the ATM (ataxia-telangiectasia mutated) kinase. This serine-threonine kinase is a major transducer of DNA damage signaling [28]. ATM activates the p53 DNA damage response by phosphorylating both Mdm2 and p53 [29], [30], and we have previously used the ATM-Ser1981 antibody as a robust indicator of DNA damage and auto-phosphorylated ATM in mice [29]. Antibody staining for phospho-S1981 ATM (activated ATM) was performed on skin samples taken from adult K5-Cre+, control mice or from the skin of age-matched Dicer^Δ/Δ^ mice and Dicer^Δ/Δ^, p53^Δ/Δ^ mice. The results (Fig. 4C) parallel what was seen in the phospho-H2A.X stains: large amounts of phospho- S1981 ATM was specifically present in Dicer-ablated skin, indicating that loss of Dicer induces DNA damage and activation of DNA damage signaling (ATM auto-phosphorylation) in the epidermis of mice. In addition, the overall level of DNA damage appears to be more profound in the skin of Dicer^Δ/Δ^, p53^Δ/Δ^ mice than in Dicer^Δ/Δ^ mice. To quantitate DNA damage levels in the epidermis, the total number of nuclei and the number of phospho-H2A.X positive-stained nuclei present in ten high-power (40X) fields were counted for representative mice of each genotype. The average percent of all nuclei showing DNA damage for each genotype is presented in Fig.4 D. Less than 1.5% of the nuclei of WT control or K5-Cre control epidermis stain positive for phospho-H2A.X. In contrast, Dicer-ablated epidermis (7.7%) and Dicer, p53 co-ablated epidermis (11.9%) display increased numbers of DNA damaged nuclei.

**Figure 5:**
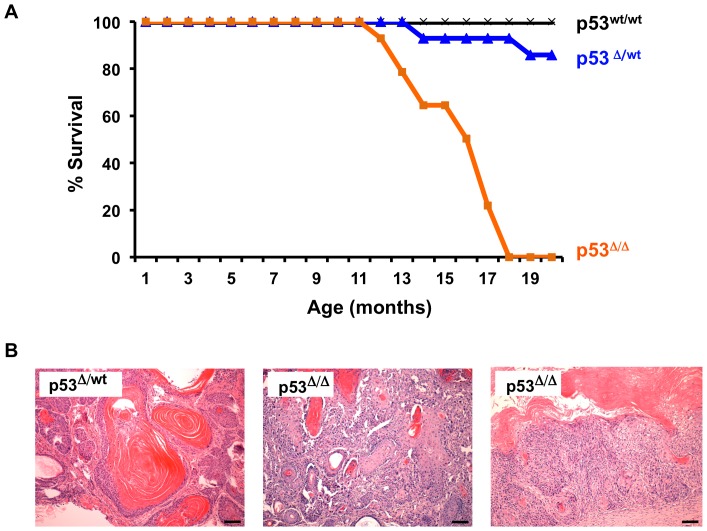
Spontaneous skin tumorigenesis in p53-deficient mice. **A**) Kaplan-Meier survival curve for K5-Cre+ mice bearing one (n = 14), two (n = 14), or no (n = 18) ablated alleles of p53 (blue, orange and black lines, respectively). Survival of p53-wildtype mice was not compromised, whereas loss of one or both alleles of *p53* reduced survival. Log-rank test indicates p53 skin-ablated mice die significantly faster than p53 skin-haploinsufficient mice or p53 wildtype mice (P<0.01). **B**) Tumor formation in mice deleted for one or both alleles of *p53*. Left panel is cystic tumor formed on ear of p53^wt/Δ^ mouse, middle panel is basal cell carcinoma formed on the face of a p53^Δ/Δ^ mouse, right panel is a representative well-differentiated squamous cell carcinoma formed on the flank of a p53^Δ/Δ^ mouse. Scale bars equal 100 microns.

Dicer^Δ/Δ^ mice retaining functional p53 alleles did not form skin tumors, whereas numerous skin tumors developed in Dicer^Δ/Δ^, p53^Δ/Δ^ mice. To confirm that these tumors did not arise solely due to loss of p53 in the skin, we performed a 20-month survival assay on K5-Cre+, p53^wt/c^ mice and K5-Cre+, p53^c/c^ mice. Two of fourteen K5-Cre+, p53^wt/c^ mice died by 20 months of age, with a single mouse developing a spontaneous cystic skin tumor at 20 months. The other mouse died from conditions unrelated to cancer (malocclusion). In contrast, all fourteen K5-Cre+, p53^c/c^ mice died by 20 months (Fig. 5A). Four mice in this cohort died from conditions unrelated to cancer (two from wounds suffered from fighting, two for unknown reasons), whereas the remaining ten mice p53 ablated for p53 in the skin formed spontaneous carcinomas. In contrast, no K5-Cre+, p53^wt/wt^ mice died during this time period (p<0.0001). Half of the p53^Δ/Δ^ tumors were well-differentiated squamous cell carcinomas (SCC), three were moderately differentiated SCC, and two tumors were scored as poorly differentiated SCC (Fig. 5B and Table 1).

**Table 1:** Tumor spectrum of mice deleted for *p53* and/or *Dicer* in skin.

Genotype of skin	# Mice	# Tumor	Classification	Multiple tumors
Dicer^wt/wt^, p53^wt/wt^	18	0	No tumors	None
Dicer^wt/wt^, p53^wt/Δ^	12	1	1 cystic mass	None
Dicer^wt/wt^, p53^Δ/Δ^	14	10	5 SCC (wd)	2 (20%)
			3 SCC (md)	
			2 SCC (pd)	
Dicer^Δ/Δ^, p53^wt/wt^	12	0	No tumors	None
Dicer^Δ/Δ^, p53^wt/Δ^	9	9	5 BCC-nodular	2 (22%)
			1 SCC (md)	
			1 anaplastic Carc	
Dicer^Δ/Δ^, p53^Δ/Δ^	25	25	1 cystic tumor	9 (41%)
			1 SCC, *in situ*	
			1 SCC (wd)	
			4 SCC (md)	
			3 SCC (pd)	
			9 invasive Carc. (vpd)	

A majority of tumors forming in the mice were fixed and classified by morphology after sectioning and staining with hematoxylin and eosin. The number of mice in cohort and the number of mice presenting with one or more tumors are given in second and third columns. Tumors undergoing histopathologic analysis and classified are given in fourth column, as are the numbers (and percentages) of mice presenting with multiple, simultaneous tumor formations (last column). SCC = squamous cell carcinoma, BCC = basal cell carcinoma, Carc = carcinoma, wd = well differentiated, md = moderately differentiated, pd = poorly differentiated, vpd = very poorly differentiated-undifferentiated.

**Figure 6:**
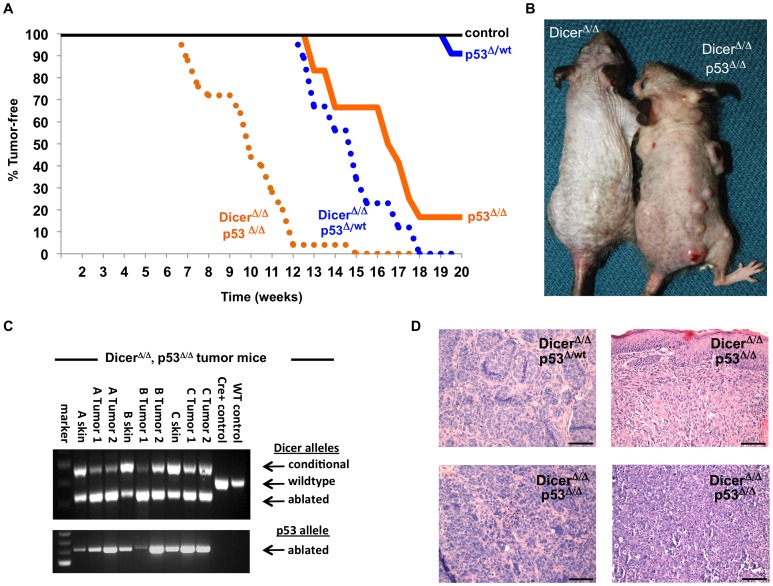
Dicer and p53 cooperate to suppress skin carcinogenesis. **A**) Kaplan-Meier tumor curves for various mouse cohorts. K5-Cre+ mice that bear *Dicer* and *p53* wildtype alleles (n = 18) do not present with spontaneous tumors (black curve). Tumorigenesis in K5-Cre+ cohorts that contain one ablated allele of *p53* (blue curves), with (n = 13) and without (n = 12) *Dicer* co-ablation, or in K5-Cre+ cohorts that bear two ablated alleles of *p53* (orange curves), with (n = 25) or without (n = 12) *Dicer* co-ablation reveals that loss of Dicer (Dicer^Δ/Δ^) accelerates cancer formation in p53-haploinsufficient mice (p<0.0001) and p53-ablated mice (p<0.0001). **B**) Representative Dicer^Δ/Δ^, p53^Δ/Δ^ mouse (on right) presenting with multiple incidence of tumor formation. Dicer^Δ/Δ^ mouse (on left) is an age-matched, 12-month old mouse with wildtype *p53* alleles. **C**) PCR analysis of tumor-derived and tissue-derived genomic DNA samples from representative Dicer^Δ/Δ^, p53^Δ/Δ^ mice reveals the presence of the Dicer-ablated allele and p53 ablated allele in the skin and tumor of the mouse, but not in control mice (right panels). **D**) Hematoxylin and eosin staining of skin tumors arising in *Dicer*-ablated mice deleted for one (p53^Δ/wt^) or both copies (p53^Δ/Δ^) of p53. Top left panel is a BCC. Mice co-deleted for both *Dicer* and *p53* form moderately or poorly differentiated SCC (top right panel) and poorly differentiated and invasive carcinomas (bottom two panels). Scale bars equal 100 microns.

Interestingly, mice ablated for *Dicer* and haplo-insufficient for *p53* in the skin died at a similar rate as Dicer^Δ/Δ^ mice during the first year. As with Dicer^Δ/Δ^ mice, the early deaths in Dicer^Δ/Δ^, p53^wt/Δ^ mice appear unrelated to cancer, as no tumors were detected in any tissues of these mice (Fig. 6A). However, members of the Dicer^Δ/Δ^, p53^wt/Δ^ colony that survived beyond one year of age developed skin tumors that accounted for the diminished long-term survival rate of this cohort relative to p53^wt/Δ^ mice (p<0.0001). Strikingly, all of the Dicer^Δ/Δ^, p53^Δ/Δ^ mice developed skin tumors by 12–15 months of age (Fig. 6A), by which time when the p53^Δ/Δ^ cohort is just beginning to show onset of skin tumorigenesis (p<0.0001). Thus, the greatly accelerated demise of Dicer skin-ablated mice when co-ablated for p53 in the skin (see Fig. 3G) is due to rapid formation of skin tumors in this cohort. Furthermore, while less than a quarter of the p53^Δ/Δ^ mice or Dicer^Δ/Δ^, p53^wt/Δ^ mice developed more than a single tumor in the skin, forty-one percent of mice co-ablated for Dicer and p53 in the skin developed multiple simultaneous tumors (Fig. 6B and Table 1).

Analysis of several Dicer^Δ/Δ^, p53^Δ/Δ^ mice forming multiple tumors confirmed the presence of the *Dicer*-ablated alleles and *p53*-ablated alleles in the skin and in tumor tissues (Fig. 6C). Histological analysis of the tumors in the different cohorts also indicated that co-ablation of *Dicer* and *p53* also led to the formation of more aggressive and invasive cancers (Fig. 6D and Table 1). Mice deleted solely for *p53* in skin formed mostly well-differentiated or moderately differentiated squamous cell carcinomas (SCC), whereas mice deleted solely for *Dicer* in skin failed to form any tumors. In contrast, mice deleted for *Dicer* and haplo-insufficient for *p53* formed mostly basal cell carcinomas (Fig. 6D -top left panel). However, mice co-deleted for both *Dicer* and *p53* formed far more moderately or poorly differentiated SCC (Fig. 6D, top right panel), and nearly half developed poorly differentiated carcinomas. These poorly or undifferentiated Dicer^Δ/Δ^, p53^Δ/Δ^ carcinomas were also highly invasive, displaying small islands and cords of tumor cells invading through dermal collagen with associated desmoplastic stromal reactions (Fig. 6D -bottom panels).

## Discussion

Previous analysis of the role of miRNA in mouse skin development has determined that prenatal deletion of either *Dicer* or *Dcgr8* grossly alters hair follicle formation and establishment of the hair follicle bulge stem cell compartment [18]–[20]. In contrast, inducible deletion of *Dicer* or *Drosha* in the epidermis in post-natal mouse skin did not cause histologic abnormalities or a loss of stem cells in resting (telogen-phase) hair follicles, but led to DNA damage and cell death in rapidly proliferating follicular matrix cells during the anagen stage of a depilation-induced hair cycle [21]. Interestingly, deletion of miRNA processing enzymes in mouse skin was also found to increase the rate of epithelial cell proliferation [18], [21]. We have previously determined that loss of miRNA biogenesis induces DNA damage and p53 activation in fibroblasts, and that ablation of *Dicer* in the follicular epithelium of young mice induces senescence in hair follicle matrix cells [6]. Interestingly, newborn mice deleted for *Dicer* due to presence of a K5-Cre transgene are not initially furless, as was the case in the K14-Cre driver models [18], [19]. Rather, K5-Cre+, Dicer^c/c^ mice develop a full fur coat that is lost post-weaning. This difference is likely due to the later onset of Cre expression in K5-Cre+, Dicer^c/c^ mice. In mice, hair cell progenitors are established during development at E14–E15, coincident with the expression of Cre in K14-Cre mice. However, the K5-Cre transgene does not promote Cre expression and Dicer ablation until just prior to birth [25], [27]. Since the epidermal stem cell compartment is properly established prior to loss of *Dicer* in our K5-Cre+, Dicer^c/c^ model, hair follicles undergo normal invagination and a fur coat is established in these mice at postnatal day 5-6. However, once the established hair follicles undergo one or two normal anagen cycles, the hair matrix is depleted and cannot be re-established by progenitors from the bulge. Thus, the Dicer^Δ/Δ^ mice lose their fur and whiskers by 6-8 weeks of age, and remain hairless throughout their life. The loss of a functional stem cell niche and normal hair cycling in the Dicer^Δ/Δ^ mice results in degraded and reduced numbers of follicles present in the skin of older mice.

### MicroRNA biogenesis and DNA damage

Although we have previously noted increased senescence in hair follicle bulge cells of young Dicer^Δ/Δ^ mice, it is unlikely that p53-mediated senescence underlies the defect in maintenance of normal hair follicles, as co-ablation of p53 in the epidermis failed to alter the rate of fur loss in *Dicer* skin-ablated mice or permit the Dicer^Δ/Δ^ mice to regrow fur later in life. However, co-ablation of *p53* did reduce the amount of apoptosis observed in the epidermis of Dicer^Δ/Δ^ mice as they aged, and resulted in an increase in cellularity of the basal and surface epidermis. Interestingly, increased amounts of phospho-H2A.X were also detected in the skin of Dicer^Δ/Δ^ mice, in keeping with our previous observations in Dicer–ablated MEFs and in recent findings of DNA damage in the skin of adult mice temporally ablated for *Dicer* [6], [21].

We have previously determined that activated ATM phosphorylates Mdm2 to regulate p53-induced apoptosis in mice following DNA damage [29], and that Mdm2-p53 signaling regulates maintenance of the murine epidermal stem cell niche [25]. In our present study, DNA damage was detected in the skin of Dicer^Δ/Δ^ mice irrespective of p53 status, and staining for activated (auto-phosphorylated) ATM revealed induction of the DNA damage response in these cells. However, apoptosis was clearly elevated in the skin of Dicer^Δ/Δ^ mice retaining functional p53, whereas DNA damage and epithelial proliferation was apparent in the skin of Dicer^Δ/Δ^, p53^Δ/Δ^ mice and in Dicer^Δ/Δ^ mice. Therefore, we conclude that loss of *Dicer* in the skin promotes DNA damage and activates the ATM-Mdm2-p53 signaling axis to induce p53-mediated apoptosis and tumor suppression in the epidermis.

It is presently unclear why loss of miRNA biogenesis triggers DNA damage in the epidermis. Since miRNAs are known to regulate the expression of oncogenes such as *Ras* and *c-Myc*, it is possible that inappropriate oncogene activation underlies the increased Ki-67 staining and proliferation of epidermal cells and results in concomitant DNA damage observed in the skin of Dicer^Δ/Δ^ mice. Alternatively, loss of *Dicer* and mature miRNAs may be altering DNA repair pathways in these cells. Further studies are needed to determine the mechanistic role of miRNA molecules in protecting epidermal cells from DNA damage.

### Suppression of spontaneous skin cancers by p53

The tumor-suppressing effects of p53 have been firmly established in mice, and mice haplo-insufficient or devoid for functional *p53* alleles develop an array of spontaneous tumors [31]–[33]. However, spontaneous epithelial carcinogenesis is rare [34]. This is likely due to the more rapid onset of lymphomas and sarcomas in these models. Chemical or UV-induced tumor studies in p53-deficient mice have determined that DNA damage can promote the initiation or progression of carcinogenesis in the skin in a p53-dependent manner, but surprisingly little data is present in the literature on the effect of p53 ablation and spontaneous tumorigenesis in the skin. Our data indicates that loss of both alleles of *p53* can promote the formation of squamous cell carcinomas in the skin of mice older than one year of age, with approximately two-thirds of the p53^Δ/Δ^ mice developing SCC by two years of age. These tumors mostly arose at a single site on the mouse, and most were characterized as having a moderately or well-differentiated morphology.

### Dicer and p53 cooperate to suppress skin carcinogenesis

*Dicer* ablation was not found to be tumorigenic in the presence of functional p53, although the early lethality of this model precludes a long-term tumor assay. However, loss of Dicer greatly accelerated tumor formation in mice co-ablated of one or both functional alleles of *p53*, and nearly all Dicer^Δ/Δ^, p53^Δ/Δ^ mice presented with skin tumors by one year of age, a time point prior to formation of any tumors in p53^Δ/Δ^ mice. Dicer^Δ/Δ^, p53^Δ/wt^ mice formed mostly basal cell carcinomas, and only a few mice developed more than a single spontaneous tumor. In contrast, Dicer^Δ/Δ^, p53^Δ/Δ^ mice developed more aggressive cancers that were often poorly differentiated SCC or undifferentiated carcinomas, and these latter cancers appeared highly invasive. Furthermore, many (41%) of these mice developed cancers at more than a single site, often developing numerous cancers that arose simultaneously in the skin. Thus, Dicer functions as a tumor suppressor in skin by cooperating with p53 to suppress carcinogenesis.

These data establish a role for miRNA biogenesis in regulating the formation of BCC and SCC in mice. Since Dicer and miRNA biogenesis functions to prevent DNA damage and excess cell proliferation in the epidermis, the Dicer^Δ/Δ^, p53^Δ/Δ^ model should facilitate the subsequent identification of specific miRNA(s) responsible for suppressing the accumulation of DNA damage in the epidermis and skin carcinogenesis.
